# Curative Treatment of Bacterial Diseases (so-called)

**Published:** 1891-06

**Authors:** Samuel Swan

**Affiliations:** New York


### CURATIVE TREATMENT OF BACTERIAL DISEASES (SO-CALLED).

Samuel Swan, M. D., New York.

In a very interesting article in the London *Lancet* of January 31st, by Dr. Sheridan Delépine on "Bacterial Diseases," the effort appears to be made to explain the preventive, protective, and curative methods of such diseases.

"1. The *preventive method* consists in *destroying* or *attenuating the cause*, or avoiding it in some way or other so that the body may remain unaffected. (a) *The antiseptic method* introduced by Lister is a good instance of the methods which aim at *destroying the cause* before it has acted. (b) *Residence* in high localities, *drainage*, etc., are instances of the methods by which the causes of disease may be so *attenuated or diluted* as to become harmless. (c) *Absolute cleanliness. Aseptic* methods are based on the possibility of avoiding certain causes entirely without destroying them.

"2. *Protection* consists in so *modifying the possible host* as to render it able to resist the virulent parasites. This can be done either by (a) increasing its *strength* and activity, as by diet, warmth, functional activity, and other hygienic conditions. (b) Rendering its tissues and fluids *unsuitable media* for the *growth* or *full development* of the parasite. *Inoculation* and *Jenner's vaccination* are good instances of that method, which has been further extended by Pasteur and others; (c) by establishing *tolerance.*

"3. The *curative methods* consist in *attenuating* or entirely destroying the virus causing the disease, *after it has penetrated into the body.* (a) The *actual destruction of the parasite within its host* is apparently still a desideratum. (b) *Attenuation of the virulence* can be obtained by introducing into the blood and tissues some product either interfering with the full development of the parasite, or modifying the tissues and fluids of the body so as to increase their resistance to the extension of the parasite or to its products. This seems to be the chief principle at the root of Pasteur's *vaccination* for hydrophobia, etc. (c) *Neutralizing the physiological action of the virus by using its physiological antagonist.* (d) *Destroying and removing the substratum* or ground which has become contaminated by the parasite. This is *apparently the view which Koch has taken of the action of his 'Lymph.'* He states his 'object in the paper was less to give an account of any single method than to trace the development of the ideas which are at the basis of the treatment of bacterial diseases. In this way I hope I may have been able to show you how *science* prepares the way for the highest branches of the *art*, viz.: *preventive*, protective, and *curative* medicine.' "

In discussing this subject, let us inquire into the cause of these so-called bacterial diseases. Is the disease caused by bacteria, or are the latter a result of the disease? Certain it is that when disease invades the organism, bacteria are discovered that are attendant in that especial disease *after* incubation. There are myriads of infinitesimals that inhabit the fluids, and their use is to destroy any peccant matter in the blood, and they have hard work to do when Koch or Pasteur injects poisoned baccilli into the blood. When the "virus causing disease" (as the above writer states it) invades the system, those parasites in the blood (not belonging to the regular army) that are in affinity with the poison have a "high old time" gorging and fattening themselves with the poison, thereby becoming objective, and are hailed as the cause of the disease.

According to esoteric science, in the far-away time called the Silver Age, these infinitesimals were strength, gentlenesses, kindnesses, or life-giving productivities—there were no parasites. These, from some occult cause, came later, and invaded the red and white fluids; "those in the white fluids being forms of ferocities." The infinite smallness of these infinitesimals enabled them to pass freely through the sheaths of the vessels and tissues. The invading poison comes first in the white or nerve fluids and thence to the red or blood fluid. All the infinitesimals each in their separate use unite to destroy the invading poison, and by absorbing it, change its nature and thus weaken it, so that in many cases the vital force is able to overcome it, and the patient recovers.

As it is well known that if two similar currents of electricity of equal power are started at the same moment from each end of a wire, they neutralize each other, so if any drug produces a poison similar to that which invades the organism, and is given to the individual, it neutralizes the action of the invading poison, and health results. In order to ascertain how a drug can be selected for the case of sickness, we must note the effects of the invading poison—that is, the symptoms produced in the organism. Then a drug which will produce a similar group of symptoms in a healthy person, if potentized by attenuation, and a dose given to the patient neutralizes the poison, as illustrated by the opposing currents of electricity. These results will be better obtained if hygienic measures are enforced, but even when they are not obtainable the result will be the same but not so quickly.

If such a remedy be given it cures by neutralizing the poison that *caused* the disease, and the parasite, the *result* of the poison disappears, probably eaten up by some of the innumerable families.

If we can realize in our thought that the disease causing poison is an immaterial aura, undetectable until it has made itself manifest in the system after a period of incubation, and that the objective bacteria are the result of that poison in the system, we can see that our endeavor should be made to antidote the poison, rather than killing the microbes. They are doing a legitimate work, helping nature, or the vital force, in ridding the system of the poison. These infinitesimals are filled with the poison, and if that can be secured, the remedy is in our hands for the cure of the disease. This can be done by taking the morbose matter and attenuating it in a definite proportion of water till it has become potentized, and all the peccant matter has disappeared, and the poison is in the potentized preparation, and so undetectable by any analysis except that of the most sensitive of all tests, the human organism.

That the presence of the microbe is not necessary to convey the poison is shown in a crude way by using Koch's Lymph or Pasteur's hydrophobic preparation. When it has settled, and the clear liquid injected, the same results follow, while the most powerful microscope can discover no microbes. Preparations potentized as above do not need to be administered by the barbarous method of injections, but are more effective when taken on the tongue. This method of cure is also the preventive, and is used successfully in small-pox, diphtheria, scarlet fever, tuberculosis, erysipelas, syphilis, glandular diseases, leprosy, scrofula, etc., etc.

If all the hard students and able writers would rid themselves of the microbe necessity, and turn their attention to antidoting the poisons that cause the disease, destructive epidemics would soon succumb to the power of the neutralizing antidote and "the actual destruction of the parasite within its host" would be the result of destroying the poison on which it feeds.

